# Improvement of Disease Resistance in Livestock: Application of Immunogenomics and CRISPR/Cas9 Technology

**DOI:** 10.3390/ani10122236

**Published:** 2020-11-28

**Authors:** Md. Aminul Islam, Sharmin Aqter Rony, Mohammad Bozlur Rahman, Mehmet Ulas Cinar, Julio Villena, Muhammad Jasim Uddin, Haruki Kitazawa

**Affiliations:** 1 Department of Medicine, Faculty of Veterinary Science, Bangladesh Agricultural University, Mymensingh 2202, Bangladesh; aminul.vmed@bau.edu.bd; 2 Food and Feed Immunology Group, Graduate School of Agricultural University Science, Tohoku University, Sendai 980-8572, Japan; jcvillena@cerela.org.ar; 3 Livestock Immunology Unit, International Research and Education Centre for Food and Agricultural Immunology (CFAI), Graduate School of Agricultural Science, Tohoku University, Sendai 980-8572, Japan; 4 Department of Parasitology, Faculty of Veterinary Science, Bangladesh Agricultural University, Mymensingh 2202, Bangladesh; s.a.rony@bau.edu.bd; 5 Department of Livestock Services, Krishi Khamar Sarak, Farmgate, Dhaka 1215, Bangladesh; mbozlur@gmail.com; 6 Department of Animal Science, Faculty of Agriculture, Erciyes University, 38039 Kayseri, Turkey; mucinar@erciyes.edu.tr; 7 Department of Veterinary Microbiology & Pathology, College of Veterinary Medicine, Washington State University, Pullman, WA 99164, USA; 8 Laboratory of Immunobiotechnology, Reference Centre for Lactobacilli, (CERELA), Tucuman 4000, Argentina; 9 School of Veterinary Science, Gatton Campus, The University of Queensland, Brisbane 4072, Australia

**Keywords:** next generation sequencing, transcriptomics, bioinformatics, genome editing, disease resistance, livestock

## Abstract

**Simple Summary:**

Disease resistance is the ability of animals to inhibit the growth of invading pathogens within the body, which is influenced by the interaction of the host immune system, host genetics, and the pathogens. Resistant animals can be produced by molecular breeding by introducing the genomic marker responsible for disease resistance or immunocompetence. Immunogenomics is an information science that enables the genome-scale investigation of host immune response to pathogenic infection thereby identification of the genomic marker for disease resistance. Once the genomic marker is determined, it could be implemented in producing disease resistance animals by applying the advanced reproductive biotechnology like genome editing. The technical ease and decreasing cost over time might enhance the application of genome editing techniques for producing disease resistance livestock.

**Abstract:**

Disease occurrence adversely affects livestock production and animal welfare, and have an impact on both human health and public perception of food–animals production. Combined efforts from farmers, animal scientists, and veterinarians have been continuing to explore the effective disease control approaches for the production of safe animal-originated food. Implementing the immunogenomics, along with genome editing technology, has been considering as the key approach for safe food–animal production through the improvement of the host genetic resistance. Next-generation sequencing, as a cutting-edge technique, enables the production of high throughput transcriptomic and genomic profiles resulted from host-pathogen interactions. Immunogenomics combine the transcriptomic and genomic data that links to host resistance to disease, and predict the potential candidate genes and their genomic locations. Genome editing, which involves insertion, deletion, or modification of one or more genes in the DNA sequence, is advancing rapidly and may be poised to become a commercial reality faster than it has thought. The clustered regulatory interspaced short palindromic repeats (CRISPR)/CRISPR-associated protein 9 (Cas9) [CRISPR/Cas9] system has recently emerged as a powerful tool for genome editing in agricultural food production including livestock disease management. CRISPR/Cas9 mediated insertion of *NRAMP1* gene for producing tuberculosis resistant cattle, and deletion of *CD163* gene for producing porcine reproductive and respiratory syndrome (PRRS) resistant pigs are two groundbreaking applications of genome editing in livestock. In this review, we have highlighted the technological advances of livestock immunogenomics and the principles and scopes of application of CRISPR/Cas9-mediated targeted genome editing in animal breeding for disease resistance.

## 1. Introduction

Foods from the livestock are a vital source of high-quality protein. Efficient production of animal-originated food is one of the major issues for global food safety, a long desire for a healthy population. The occurrence of infectious diseases in livestock affects not only the farm production, economics, and animal health-welfare; but it also increases the risk of zoonoses. Therefore, the outbreak of infectious diseases in food animals is a major threat to food safety and public health. The emergence of antimicrobial drug resistance along with the unavailability of effective vaccines and spontaneous genetic mutation of infectious pathogens are the major perils for breaking down the disease control strategies [1]. On the other hand, there is a growing consumer demand to produce organic animal food by securing animal welfare standards. Raising disease-free healthy livestock is the key to produce safe meat and milk. Good husbandry practices including optimum housing, ration, use of probiotics, biosecurity, and vaccination should be taken into account for the maintenance of health and production of farmed animals [2]. However, the improvement of host genetic resistance to disease may potentially contribute to the profitability through improved animal welfare and reduced antibiotic usage in livestock production [3]. 

**Figure 1:**
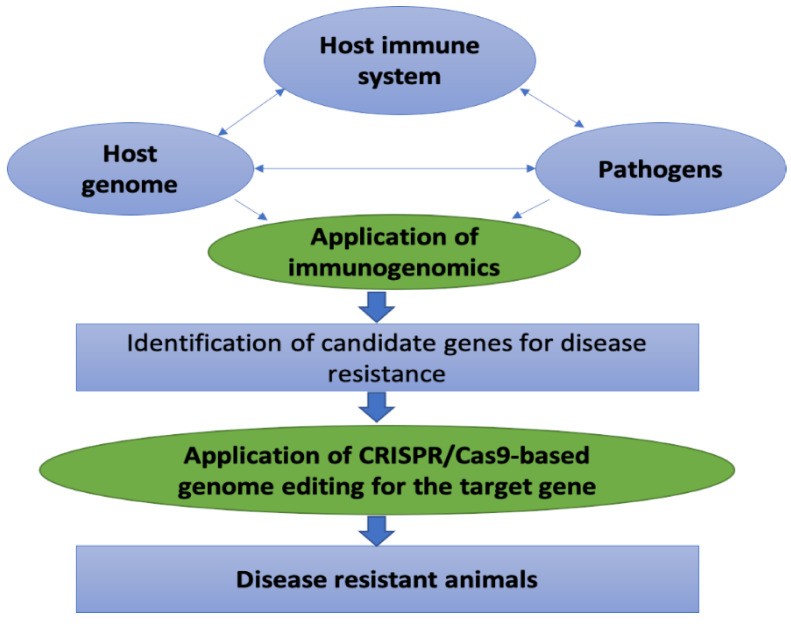
Schematic diagram showing the applications of immunogenomics and genome editing to produce disease-resistant animals. Severity and pathogenesis of disease depend on the interaction of the host immune system and the invading pathogens, where host genetic has potential influence. Immunogenomics employ the integrated bioinformatics tools to explore the influence of host genetic on the interaction between the host immune system and invading pathogens and subsequently identify the candidate gene (s) for disease resistance. The CRISPR/Cas9 mediated genome editing technology could subsequently be employed for targeted modification of the host genome to produce disease-resistant animals.

Disease resistance is the host’s ability to restrict or inhibit the establishment of infections and/or pathological processes of infectious pathogens [4]. Interactions among the host immune system and invading pathogens, and host genome determine the fate of infection and disease pathogenesis (Figure 1). Innate immunity is the frontline host defense against invading pathogens, and genes associated with the induction of innate immune responses are considered as the potential candidates for disease resistance [5]. Innate immune responses provide immediate and non-specific defense against a wider range of pathogens; therefore, innate immune response traits are the first choice to be incorporated in the disease-resistant breeding plans. Notably, the heritability of the innate immune response is medium to moderate [6]. The contribution of host genetic factors to disease occurrence is one of the fundamental issues in understanding disease pathogenesis and host resistance. The variation in the genetic resistance to disease is due largely to the variability in the host’s immune response to infection. Thus, information on immunology and genomics together would better characterize the disease phenotype [4]. The application of genomic technologies to understand the immunology is termed as immunogenomics. Immunogenomics include integrated analysis of immunologic and genomic data on host response to infectious pathogens, and thereby contribute to identifying potential candidate genes for disease resistance in livestock [7]. The single nucleotide polymorphisms (SNPs) within the candidate gene associated with host immunocompetence to infection can subsequently be considered as a DNA marker for disease resistance.

Genome editing is a bio-engineering technology that involves insertion, deletion, or modification of a specific section of DNA sequence in the genome. The genome-editing technology encompasses nuclease enzyme for cutting DNA sequence, in addition to a targeting mechanism that guides the enzyme to a particular site on the genome [8]. The clustered regulatory interspaced short palindromic repeats (CRISPR)/CRISPR-associated protein 9 (Cas9) [CRISPR/Cas9] system is one of the latest genome-editing tools that has been widely used over the last couple of years (reviewed by Pellagatti et al. [9]). Genome editing in livestock has become a commercial reality due to the emergence of CRISPR/Cas9 technology. Advance use of CRISPR/Cas9 could facilitate the improvement of disease resistance in livestock through (a) enhancing the frequency of favorable trait-associated alleles, (b) introgression of favorable alleles from other breeds/species, and (c) by generating de novo favorable alleles [10]. However, the main challenge is the identification of genome-editing targets for a disease-resistant trait, which will require a combination of high-quality annotated livestock genomes, well-powered genome-wide association studies, and robust knowledge of molecular genetics of pathogen-host immune system interactions. In this review, we have systematically explained how the gift of immunogenomics could be applied to grasp the disease resistance candidate genes and use of the biological scissor, CRISPR/Cas9 technology, for insertion or deletion of desired genes in the host genome (Figure 1). The prospects, regulations, and social acceptance of genome editing technology concerning improving the livestock health and production are also discussed.

## 2. Disease Resistance: The Phenotype

Host-pathogen interactions result in either death or survival of the affected animal. Survivors either remain healthy and unaffected by the pathogens or experience a course of a disease that recover afterward. The immune system plays a crucial role in maintaining a balance between health and disease pathogenesis. In general, disease resistance demonstrates the host’s ability to limit or prevent the replication of invading pathogens [11,12], and the relationship between the host and pathogens is better understood from an ecological point of view [13]. This concept includes several ways through which a host becomes comparatively more robust. For example, to display less possibility of infection, to have a reduced proliferation rate once infected, to possess a reduced rate of shedding or transmission [4]. It is not easy to measure the disease resistance trait, which is a major challenge for the investigator. It is possible that phenotype of disease resistance is relative rather than absolute and altered resistance impacts on the population as a whole because few attributes favor the individual host in certain cases, but other attributes (such as lower rate of transmission and infection) favor other population members. There is another associated phenotype called disease resilience, the capacity of the host to suppress the establishment or development of infection. Disease resilience is a physiological state in which an infected animal is capable of sustaining an acceptable degree of efficiency despite the burden of the pathogen [14]. The prospects and possibilities of using disease resilience in animal breeding programs are reviewed by Berghof et al. [15]. In addition, tolerance is another closely related phenotype that indicates the ability of the host to maintain the body homeostasis in the presence of replicating pathogens, with limited pathological consequences [12]. In a mixed population, the presence of asymptomatic and disease tolerant individuals may increase the genetic resistance of individual hosts, but there is a risk of increasing the prevalence of disease on the farm. Therefore, the disease resistance phenotype could consider to be incorporated into the host genetic improvement strategy. 

Absolute quantification of the disease resistance phenotype under field condition is very difficult due to logistic and economic constraints, which is one of the major rate-limiting steps in animal breeding for disease resistance. Therefore, targeting immunocompetence trait is the indirect approach for improving disease resistance in livestock, and thereby producing safe milk and meat in the age of antibiotic resistance. Immunocompetent animals possess higher metabolic function and resilience to a wider range of infectious diseases, thereby improving the production performance in terms of quality and quantity. The host resistance to infectious diseases in livestock could be enhanced by incorporating resistance genes in the host genetics. The most economically important diseases in different livestock species could be considered first in the breeding goals for more sustainable production of disease-resistant animals [3]. Some infectious diseases of economic importance are mastitis, foot and mouth disease (FMD), tuberculosis, John’s disease, and tick-borne diseases in cattle, Peste des petits ruminant (PPR) in goats, porcine reproductive and respiratory syndrome (PRRS), African swine fever in pigs, and gastrointestinal (GI) parasite infection in cattle and sheep [16]. 

Breeds of different animal species have a great influence on the innate resistance to infectious disease. Native breeds usually exert a higher degree of resistance to pathogens than the high-yielding exotic breed of the same species, which is believed to be due to the baseline immune competence, mainly innate immunity which could be transmitted down the progeny via genetic information and colostrum. Indigenous breeds of cattle (*Bos indicus*) were found to have a higher resistance to tick infestation and tick-borne diseases compared to high-yielding crossbred cattle as evidenced by higher hemolytic complement activity [17]. Native breed’s resilience to local pathogens could be gained through evolution and adaptability when raised in the extensive farming system [18]. On contrary, intensive farming may weaken hosts’ innate immunity. The relative difference in immunocompetence to infectious disease was reported among porcine breeds [19,20]. The genetic components associated with disease resistance should incorporate into the breeding program. Advances in tools and techniques for studying immunology and genetics of livestock and data analyses by immunogenomics enabled animal scientists to implement the molecular breeding technique including marker-assisted selection, genomic selection, and targeted genome editing for sustainable livestock production. 

## 3. Advances of Immunogenomics

Immunogenomics is an information science that deals with big data from immunology and genomics. Immunogenomics integrates the molecular interactions among the host genome, immune system, and the invading pathogens. Understanding the function of the mammalian immune system has been broadening since the intersection of immunology and high-throughput sequencing technologies. Immunogenomics combines the transcriptome, DNA variants, polymorphisms/SNPs, and quantitative trait loci (QTL) mapping data resulted from host-pathogen interactions through a series of integrated bioinformatics [7]. Thus, immunogenomics have been considered as an efficient omics tool for identifying disease resistance genes or DNA markers [7]. Transcriptome profile provides an overview of expressed genes associated with particular phenotypes and is used as guidelines for subsequent analyses by proteomics, metabolomics, epigenomics, and other omics approaches. Since transcriptome can explore the relationship between genotype and phenotype of an organism, it has been considered as the most informative assay to start with for the functional genomics. Microarray and next-generation sequencing (NGS) are the cutting-edge technologies for transcriptomics or immunogenomics in many areas of the life sciences [21]. In parallel to the revolutionary progress of NGS technology, some advanced tools for cell biology studies are equally impactful. For example, flow cytometry and mass spectrometry/cytometry can provide a better picture of the phenotypic diversity among immune cell subsets [22]. Besides, the development of the induced pluripotent stem cell (iPSC) model from peripheral T cells has opened up another exciting avenue of immunology research [23].

### 3.1. Sequencing Technology

As a cutting-edge tool for immunogenomics, the NGS platform has rapidly evolved over the past 15 years, and exponentially increased amounts of sequence data generated per instrument run at ever-decreasing costs [24]. NGS-based RNA sequencing (RNA-Seq) can quantify all sorts of RNA species including messenger RNA (mRNA), microRNA, small interfering RNA, and long non-coding RNA, which enables the researcher to discover novel RNA forms and variants. The NGS methodology has been extended through the development of single-cell RNA sequencing (scRNA-seq) and in-situ RNA sequencing [24,25,26]. A single cell is the smallest structural and functional unit of a living organism, as such a particular cell represents a specific unit of molecular coding across the DNA, RNA, and protein expression [27]. Therefore, the omics-based investigations are highly expected to be carried out at the single-cell level for more precise results. The scRNA-seq has tremendous progress in the last couple of years owing to overcoming the difficulties of isolation of a single cell population [28]. The technological progress and application of the single-cell sequencing platform about cancer research have been well-summarized in Huang et al. [28]. The scRNA-seq has recently advanced along with the development of whole-genome sequencing such as scRNA methylation sequencing and single-cell assay for transpose-accessible chromatin sequencing (Single-cell ATAC-seq) [28]. Recently, the direct RNA sequencing using high throughput nanopore sequencing technology has emerged as the latest state-of-the-art RNA-Seq technique [29]. However, the single-molecule, long-read sequencing-based NGS technology is coming soon which may replace the ongoing platforms [30].

Currently, Illumina and Thermo Fisher’s (Ion Torrent) standard and commonly used NGS platforms are based on short-read sequencing technologies [31]. To build sequencing ready libraries with an average length of 300 bp (ranging from 200–700 bp), short-read RNA-seq requires either fragmentation followed by reverse transcription or full-length cDNA synthesis followed by fragmentation. Since most mammalian RNA transcripts are 1–2 kb in length [32], getting complete RNA sequencing information relies on agreement with the annotated whole transcriptomic sequence or de novo transcriptome assembly approaches. In addition, genes have more than a transcriptional isoform that confronts the performance of the NGS in accurate gene expression quantification. The mRNA molecules transcribed from the same locus are referred to as transcriptional isoforms because mRNA can be produced from different transcriptional start sites, terminated at different polyadenylation sites, or as a result of alternative splicing [33]. Due to the limitations of short-read sequencing, it is difficult to assemble all expressed isoform for each gene and quantify the expression of all the isoforms with currently available bioinformatics tools [34,35]. To address these limitations, two commercial companies have recently launched the single-molecule, long-read sequencing technology-based NGS platform: Pacific Bioinformatics (PacBio), and Oxford Nanopore Technologies (ONT). With these technologies, the read length achieved (~15 kb for PacBio and >30 kb for ONT) exceeds the length of most mammalian transcripts. Combined with the benefit of full-length cDNA synthesis [36]. Especially SMARer (Switching Mechanism at RNA Termini) technology, long-read technologies are commercially available from Clontech (Mountain view, CA, USA), makes full-length mRNA sequencing possible with more precise transcriptomic studies. While a very powerful approach to unraveling the full spectrum of gene expression profiles is represented by long-read technologies, the relatively high costs of these technologies have prevented the broader spectrum of use. Oikinomopoulos et al. [30] reviewed the recent scientific and methodological developments in transcriptome profiling using the newly introduced single-molecule, long-read sequencing technology. 

### 3.2. Bioinformatics Tools

While in the NGS platform, sequencing has become relatively straightforward with overcoming the technological limitations, the processing of upstream samples and downstream data analyses are still labor-intensive. To make a meaningful biological analysis, big data obtained from microarray and RNA-seq experiments requires high-power statistics and rigorous bioinformatics. Although most of the RNA-Seq data analysis algorithms can be run either in a Unix environment or inside the R/Bioconductor environment [37] from a command-line interface, some web-based menu-driven interfaces (e.g., Galaxy (www.usegalaxy.org), Geneious (www.geneious.com)) also support NGS data analysis. For the identification of DNA variants/SNPs associated with features like disease resistance, NGS sequence reading has been an area of rapid development [38]. Data from DNA variants (SNPs) or genome-wide association studies can be incorporated to explore the association between genomic architecture and the traits of interest once the potential candidate genes have become available from transcriptome analysis. The Animal QTL database (QTLdb) has been developed to bridge genotypes and phenotypes by repositing all publicly accessible QTLs and SNP/gene association data on animal species [39]. Approximately 191,422 QTLs have been curated to date, including 142,261 for cattle, 30,580 for pigs, 12,246 for chicken, 3305 for sheep and 2446 for horses (https://www.animalgenome.org/cgi-bin/QTLdb/index, Release 41, 26 April 2020). In order to analyze, annotate and visualize such genomic data for complex phenotype, such as immune repertoires, including disease resistance, bioinformatics software tools are essential components [40].

The keystone of immunogenomics is data integration. Accordingly, the scientific community can benefit from data sharing strategies that facilitate the integration of datasets among research groups. However, reliable methods for data integration are needed and require a broad range of expertise such as in mathematical and statistical models, computational methods, visualization strategies, and deep understanding of complex phenotypes. The commonly used tools and databases for immunogenomics of animals are summarized in Table 1.

While in silico genomics tools and databases for the human genome have been developed well, those for the genomes of livestock are still growing. Thus, an ID conversion tool, such as bioDBnet [46], is required to convert gene ID to human orthologous identifiers to perform the downstream functional analysis using human database. BovineMine, for instance, is a useful database and web portal for data mining for the annotation of bovine genes and genomes [45]. Standardize laboratory workflows, raw data formats, experimental designs, and methods of biostatistical analyses are, however, required. The technological shortcomings of immunogenomics are being resolved and are likely to be put in the life sciences among the largest ‘big data’ enterprises [51]. The Functional Annotation of ANimal Genomes (FAANG www.faang.org) was developed in this regard to support the standard protocol for core research, data research and meta-data analysis of standards for swine immunogenomics studies [52]. In addition, the 1000 Bull Genome Project has provided the bovine research community with a huge volume of data on bovine variants that will be useful for GWAS and the identification of causal mutations (http://1000bullgenomes.com). These initiatives pave the way for a systemic incorporation of the findings of bovine immunogenomics studies and for making them available online.

## 4. Applications of Immunogenomics in Livestock Disease Management

Sustainable management of livestock diseases requires breeding techniques that protect the environment, animal welfare, and public health, as well as providing adequate financial rewards for farmers. Several efforts are in progress to develop a disease-resistant stock through molecular breeding. By dissecting the genetic makeup, a typical first stage of molecular breeding by marker-assisted selection, genomic selection, or genome editing is to determine the extent in genetic variation on the individual trait. Simple understanding of host immunology and genetics better characterize the disease-resistant phenotype. The advent of immunogenomics provides more accurate identification of candidate biomarkers for disease resistance, which makes it easier to enhance disease resistance by genome editing techniques.

In a real-life situation, the direct estimation of disease resistance phenotype is very difficult because a precise animal identification and disease regression method and infection challenge routines are required [53]. The disease resistance phenotype is typically quantified in the direct disease model by calculating the magnitude of the infection, such as burden of the inside-host pathogen (e.g., viral load), and these are technically difficult to incorporate in the intensive commercial production system. In addition, the direct measurement techniques target the host’s resistance to a particular infection. However, when introduced by molecular breeding, it can result in increased susceptibility to other infections [54]. Appropriate indirect methods are therefore highly desirable for estimating disease resistance. One method may be to estimate the phenotype of disease resistance by defining the genomic marker associated with the immunocompetence of the host produced from vaccination that is inheritable and linked to improved health and production performance [55,56]. Vaccination does not cause disease, but it allows memory T-lymphocytes, B-lymphocytes, and antibodies to be formed by the host immune system, enabling hosts to defend the subsequent infection [57]. The most suitable candidates for disease resistance traits are possibly genes regulating the first few hours of the host’s response during innate immunity to infection or vaccination [5,58]. Due to the rapid onset and wider range of defense, innate immune traits have the potentials to be used in the selection of livestock for disease resistance [53], and the innate immune traits display significant genetic variation among the breeds of livestock species [6,59]. Thus, as a possible indirect indicator of disease resistance, enhancing innate immunocompetence is of great importance.

**Figure 2:**
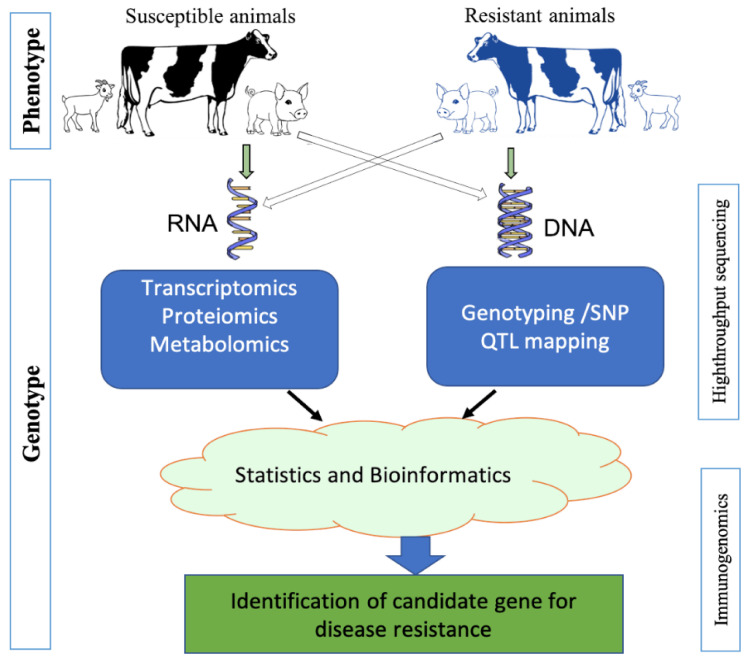
A working pipeline of (in vivo, in vitro, and in silico) immunogenomics for identification of disease resistance candidate gene/marker as prospective targets for genome editing. Isolation of single-cell population of a target from both phenotypic groups followed by RNA and DNA extraction separately. The RNA samples could be employed for proteomics and metabolomics profiling. On the other hand, DNA samples could also be subjected to single nucleotide polymorphisms (NSP) sequencing and genotyping, quantitative trait loci (QTL) mapping, and genome-wide association study, and epigenomics study targeting the disease resistance phenotype. Rigorous integrated bioinformatics application on all sets of omics data together enables us to identify the molecular biomarker for the target immunocompetence trait. After functional validation of the identified biomarkers in the independent population, those could be recommended as the targets for CRISPR/Cas9 mediated genome editing technology.

To identify the potential biomarkers for immunocompetence as indirect measures of disease resistance in livestock, the study population could be divided into two contrast phenotypes correlated to disease resistance (Figure 2). The contrast phenotypes may be achieved by either taking animals with the extreme immune response phenotype following vaccination (extremely high antibody titre vs. extremely low antibody titre) or vaccinated vs. control animals. In order to obtain adequate statistical power from the experiment, the use of an optimum number of animals as biological replicates for each group should be considered. Schurch et al. [60] proposed that for the detection of substantially differentially expressed gene between two contrast phenotypes using RNA sequencing technology, at least six biological replicates should be used. The single population of target primary cells should be separated once the experimental group is fixed in order to prevent cell-type-specific gene expression. Then, a pure single-cell population should be subjected to the extraction of total RNA and genomic DNA separately. To scan the full spectrum of gene expression between the contrast phenotypes, the quality-assured total RNA samples can be subjected to holistic transcriptome profiling by RNA-seq. The RNA samples could be used for profiling for proteomics and metabolomics. On the other hand, SNP sequencing and genotyping, quantitative trait loci (QTL) mapping, and genome-wide association study (GWAS) may also be subjected to DNA samples, and epigenomics analysis targeting the phenotype of disease resistance. Rigorous integrated bioinformatics framework on all sets of omics data together helps us to identify the molecular biomarkers for intended immunocompetence trait. Those could be suggested as the targets for CRISPR/Cas9 mediated genome editing technology after functional validation of the identified biomarkers in the independent population.

Applications of immunogenomics in human disease particularly in cancer have much progress such as the in-silico prediction of human leukocyte antigen (HLA) gene has been accelerated through a combination of high-throughput sequencing technology and specialized computational approaches [61]. Immunogenomics have been employed to explore the porcine resistance to Gram-negative bacterial infection in porcine [7] and gastrointestinal nematode infection in ruminant [62]. Knowledge of immunogenomics has been applied in the identification of several immune response genes in livestock based on their association with resistance or susceptibility in several diseases and their proven function in disease pathogenesis [16]. Several GWAS have been performed aiming to identify the QTLs or SNP profiles associated with resistance or susceptibility to bovine mastitis [63], and milk somatic cell count as an indicator of mastitis [64,65]. Many immune response genes are associated with mastitis resistance including cytokines IL-4, IL-8, IL-13, and IL-17 [66,67]. Nevertheless, further fine-tuning of the genomics tools and in silico omics software recourses in near future would enable the identification of disease resistance candidate biomarkers in a more precise way. 

## 5. Advances of Genome Editing Technology

**Figure 3:**
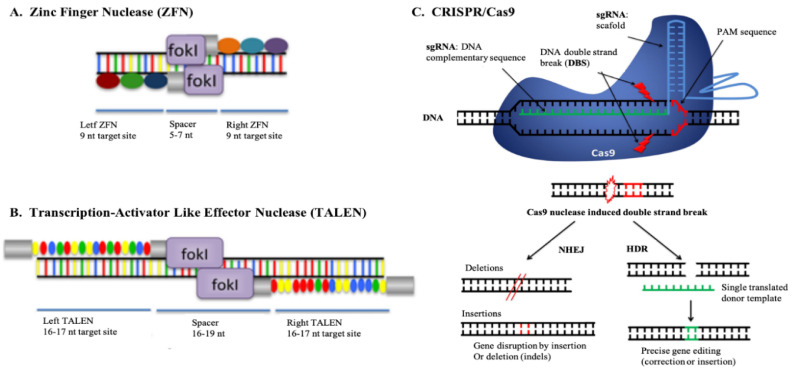
Nuclease-based genome editors. (**A**). Zinc Finger Nuclease (**B**). Transcription-Activator Like Effector Nuclease (TALEN). (**C**). Schematic diagram showing genome editing using CRISPR/Cas9 system. The Cas9 induces DNA double-strand break (DSB) which are repaired either by imperfect nonhomologous end-joining (NHEJ) to generate insertion or deletion (indels) or if a repair is provided, by homology-directed repair (HDR) (Adapted from Moore et al. [71], and Pellagatti et al. [9].

The premier seed for the possibility of a desired improvement of the mammalian genome has sown by Palmiter and Brinster in 1988 [68]. The active insertion into the mouse embryos by pronuclear microinjection of a foreign DNA fragment, a growth hormone gene called metallothionein-I, resulted in the rapid growth of the animals as targeted [68]. This technique of genome engineering based on cells was restricted to injecting plasmids or gene fragments into the embryos pronucleus. Subsequent advances in genome engineering have been achieved by the introduction of transposons or retroviral vectors [69], followed by the development of homing endonuclease (HEs), natural meganuclease capable of introducing double-strand breaks (DSBs) at 14–40 bp target sites [70]. Nevertheless, in vivo application of HE-based genome modification has been limited due to their off-target cutting propensity. In general, modern genome editing relies on DNA insertion, deletion, or substitution in the genome of a living organism using programmable nuclease-based editors (Figure 3). Zinc finger nuclease (ZFNs) [71,72] and transcription activator-like effector nuclease (TALENs) [73] were the most widely used genome editors until recently. Zinc fingers (ZFs) are among the most well-characterized DNA-binding protein domains found in the nature that have enhanced the programmed modification repertoire of enable any target genome to be accurately cut and repair [72]. A second group of naturally occurring proteins containing a DNA-binding domain and formed by proteobacteria of the genus Xanthomonas is transcription activator-like effector (TALE) [73]. A pair of ZFNs must bind regions flanking the target locus for genome editing to form a FokI dimer, which is required to induce DSBs (Figure 3A) [74]. Similarly, TALENs are also modular proteins that contain two domains: a customizable DNA-binding domain (TALE) and the FokI nuclease domain. The TALE-binding DNA sequence is cut by dimerization and thus produces DSBs in a similar manner to ZFNs (Figure 3B) [75]. However, both ZFNs and TALENs are limited by targeting multiple sites in the same genome. The comprehensive interaction of ZFNs with protein-DNA and the highly repetitive nature of TALENs warrant the advent of new instruments. 

The clustered regulatory interspaced short palindromic repeats (CRISPR)/Cas9 system, which was built from an inherent antiviral mechanism found in bacteria [76], is the latest addition to the genome toolbox. In comparison to a classical genetic modification that involves moving genes from species to another, the CRISPR/Cas9 system relies on the use of molecular ‘scissors’ to introduce changes in existing DNA sequences [75,77]. The CRISPR/Cas9 system uses a single guided RNA (sgRNA) to help the Cas9 nuclease to classify the particular genomic sequence, unlike the ZFNs and TALENs, which use proteins to recognize specific sequences in the genome (Figure 3C). In the presence of sgRNA complementary sequence and the Porto-spacer adjacent motif (PAM) sequence [9], the Cas9 protein binds to the sgRNA scaffold and generates a DSB. The DSB activates the machinery for the repair of endogenous cellular DNA that catalyzes non-homologous end joining (NHEJ) and homology direct repair (HDR) (Figure 3C). The NEHJ pathway is preferably used by most cell types, which is possibly an error-prone mechanism that generally results in minor insertions or deletions at the site of repair. NEHJ also produces a mutation and induces encoded protein fragmentation or functional gene knockout by producing DNA break in the coding sequence of a gene. On the other hand, the HDR pathway can be activated through flooding the target cell with a DNA repair template, which allows the implementation of specific sequence changes proximal to the cut site ranging from single base changes to the insertion of transgenes. In order to make minor change, a synthetic single-stranded oligodeoxynucleotide is used, whereas larger modification requires the plasmid/dsDNA template. Finally, two simultaneous breaks are produced and transcriptional profiles are altered either by removing regulatory elements or by deleting exons. Protein domains are subsequently deleted, leaving the remaining reading frame intact [78]. One of the CRISPR/Cas9 system’s key advantage is that the Cas9 nuclease is not covalently fused to a DSB, so it is possible to use same protein to attack multiple target sites by combining it with a different sgRNA package. Besides, most of the necessary reagents can be made in the molecular biology laboratory, while Cas9 nuclease and sgRNA molecules can be purchased commercially. The CRISPR/Cas9 system has made the genome-editing technique a practical reality in recent years due to its methodological simplicity, performance, precision, flexibility, and a greater degree of accuracy. 

A variety of techniques are available to produce the genetically modified animals depending on the species and cell type used to deliver the Cas9 genome-editor (reviewed by Harwood et al. [79]). The technique of sperm mediated gene transfer (SMGT) is used to deliver the genome-edited reagents into a zygote. Manipulation of spermatogonial stem cells (SSCs) or primordial germ cells (PGCs) is often used for the development of genome-edited organisms. For the development of genome-manipulated animals, the pronuclear and intracytoplasmic injection of genome editors into zygotes accompanied by either direct transfer to surrogates or in vitro maturation and embryo transfer could be applied. In addition, somatic cell nuclear transfer (SCNT) is another approach that enables precise edits to be selected in somatic cells before nuclear transfer to surrogates or in vitro maturation. The injection of edited embryonic or iPSC into blastocyst may also be used for the development of chimeric animals examined by Harwood et al. [79]. Like the genome-editor tool use, the techniques follow is also important to achieve the intended efficiency and precision in generating genome-edited animal species. The introduction of genome editing in the research animal model for the treatment of human disease has also made tremendous progress, in addition to the development of CRISPR/Cas9 techniques in animal disease management. Recent reviews have summarized the methodological advances of CRISPR/Cas-9 mediated genome editing in large animals (pigs, monkeys, dogs, rabbits, mice, rats) with a focus on the creation of model animals for studying human diseases [80,81]. 

## 6. Applications of Genome Editing in Livestock Disease Management

The CRISPR/Cas9-based genome editing has been applied in livestock for several specific (but not limited to) purposes: (a) for inactivation or alteration of expression of targeted genes in the model animals to confirm their functions (b) for producing research animals for studying human disease pathogenesis in controlled setup and evaluate potential therapies, (c) and producing genetically modified animals for industrial, pharmaceutical, and biotechnological implications [82]. Nevertheless, the success of the gene-editing system in controlling animal diseases would be affected by several factors. For instance, the proportion of gene-edited animals and how they are distributed within and across the population. According to epidemiological theory, a certain proportion of gene-edited animals required to achieve herd immunity, and to prevent disease transmission within the population [83]. Disease-specific epidemiological modeling could provide information on the required number of genome-edited animals for preventing certain disease/infectious agents. Such modeling should consider the influence of population structure, demographic characteristics, diverse environmental factors, and management strategies that affect the disease transmission dynamics to estimate the size of the population for genome editing. The latest advance in genome editing with programmable nucleases, such as CRISPR/Cas9, has opened up new avenues for animal breeding targeted with disease resistance. However, the efficiency of genome editing varies due to the variation in reproductive physiology among different livestock species. Genome editing in cattle accompanied by major challenges due to high market value, a small number of offspring, and longer gestation period (9 months gestation, 12–18 months to reach puberty). While pigs, sheep, and goats have several advantages as they are smaller, cheaper, and produce more offspring at a time, shorter gestation lengths, and shorter ages of puberty. Over time, genome editing has been successfully applied in livestock species to improve different traits like growth, production performance, and resistance to certain diseases. 

Myostatin (*MSTN)* gene is known to be associated with growth and skeletal muscle development. *MSTN* was targeted in the earlier attempts of genetic manipulation in farm animals because the disruption of this single gene has significant effects on meat production, an economically important trait. To date, genome editing has been successfully applied to knockout the *MSTN* from the genomes of cattle, pigs, sheep, and goats (reviewed by Petersen [8]). In cattle, the introduction of antibiotic lysostaphin by SCNT resulted in secretion of milk protein that has bactericidal activity against mastitis-causing bacteria *Staphylococcus aureus* [84]. The gene encoded with bovine whey protein ß-lactoglobulin (BLG), which is a major milk protein and a dominant allergen, was successfully knocked out in the bovine genome using ZFNs technique [85]. Another genome editing study has described the production of swine with mutation RELA (p56) gene using ZFNs, which confer the tolerance of African swine fever [86]. Production of a hornless strain of dairy cattle (Holstein Frisian) by inserting the pooled gene (P) of beef cattle (Angus) through the HDR pathway of gene editing has also been reported [87]. With the evolution of CRISPR/Cas9-based genome editing and a deeper understating of how to gear up its potential, it is now possible to introduce extremely precise changes to the genome with better accuracy and efficiency than any previous attempts. The CRISPR/Cas9 mediated insertion of *NRAMP1* gene for producing tuberculosis resistant cattle and deletion of *CD163* gene for producing porcine reproductive and respiratory syndrome (PRRS) resistant pigs are two groundbreaking application of genome editing technique in livestock. Hereinbelow, we summarized some prominent examples of implication of CRISPR/Cas9-based genome editing to produce disease-resistant livestock (Table 2). 

### 6.1. Porcine Reproductive and Respiratory Syndrome (PRRS) in Pigs

PRRS is considered the most economically important infectious disease of the swine industry worldwide affecting the production, reproduction, health, and welfare of pigs. The global transcriptome profiling of peripheral blood mononuclear cells revealed that pigs can induce innate and subsequent adaptive immune response to PRRS virus infection or vaccination [92,93]. The host transcriptomic response to PRRSV has been found to have substantial genetic variation [19,20]. The cluster of differentiation 163 (CD163) is a cell surface receptor gene, which is a member of scavenger receptor cysteine-rich superfamily (SRCR) having one intracellular domain and nine extracellular SRCR domains [94]. The CD163 facilitates the PRRS virus to enter into the pulmonary alveolar macrophage through endocytosis, where the virus replicates and induces disease pathogenesis [94,95]. Whitworth et al. [96] reported for the first time that CRISPR/Cas9 mediated CD163-knockout pigs were fully protected against the clinical outcome of PRRS virus infection with a single isolate [96]. A subsequent experiment demonstrated that the SRCR5 domain is crucial for establishing viral infection [97]. The implication of reproductive biotechnology for the production of genome-modified pigs might therefore significantly reduce the PRRS-associated economic losses in the pork industry.

### 6.2. African Swine Fever Resistance in Pig

Another economically relevant infectious disease caused by the African swine fever virus (ASFV) in pigs is African swine fever (ASF). Many areas of sub-Saharan Africa are endemic to the ASF. It has been detected recently in Eastern Europe, from where it is rapidly spreading to both Western Europe and China. Native wild swine breeds including the Warthog, have been reported to be resilient to ASFV infection, whereas domestic pigs experience cytokine storm-related lethal hemorrhagic fever. Significant variation in the expression of the RELA gene between resilient and susceptible pigs is associated with host response to ASFV infection [98]. The RELA is a part of the Nuclear Factor kappa Beta (NFkB) transcription factor, considered to play an important role in stress management and immune defense. By using the ZFN-based genome editing technique [99], the sequence of RELA gene in domestic pigs could be translated to that of Warthog pigs. However, the phenotypic data supporting the genetic resistance to ASFV infection yet to be reported. 

### 6.3. Tuberculosis Resistance in Cattle

Bovine tuberculosis (bTB) is a chronic bacterial disease that is crippling and caused by *Mycobacterium bovis* in cattle. With a wide host range, *M. bovis* infection create considerable hardship for livestock producers with estimates of more than 50 million cattle infected worldwide [100]. This zoonotic infection can be transmitted to humans mainly through the ingestion of milk products that are not pasteurized, resulting in a 10–15% prevalence of human TB [99]. Compulsory testing accompanied by the slaughter of test-positive animals, which accounts for as huge economic loss, is an important means of bTB regulation. The bTB is as a crucial target for genome editing for producing tuberculosis resistant cattle, due to its economic and zoonotic importance, endemic existence, and failure of conventional control strategies. As a strong candidate gene for tuberculosis resistance, natural resistance to infection with intracellular pathogens 1 (*NRAMP1*) gene has been reported by several studies. In a recent study, Cas9 nickase (nCas9) was used by scientist to insert the *NRAMP1* into the genome of the bovine fetal fibroblast [90]. These engineered fibroblast cells were then used as donor cells in somatic cell nuclear transfer, where the *NRAMP1*-containing donor cell nucleus was inserted into the cow’s ovum. Before being transferred to recipient cows following a physiologically natural estrous cycle, ova were then nurtured in the laboratory up to embryos. The inserted *NRAMP1* gene was correctly expressed and provided cattle by showing a higher degree of resistance to *M. bovis* infection [90]. It has also been reported that resistance against *M. bovis* infection could be achieved in cattle through TALEN-mediated insertion of mouse SP110 gene into an intergenic region of the bovine genome [101].

### 6.4. Enzootic Pneumonia Resistance in Cattle

Pasteurellosis in cattle also called shipping fever or enzootic pneumonia is a respiratory disease complex mostly found in recently weaned, single-sucked beef calves after housing or transport to a new house. Following infection, *P. hameolytica* secretes a leukotoxin that is cytotoxic and it binds to the uncleaved signal peptide of the CD18 protein on the surface of leukocytes. However, the mature CD18 lacks the signal peptide in the leukocytes of other species (e.g., mouse and human) that do not suffer from this disease. ZFNs have been used to introduce a single amino acid change in the bovine CD18 protein and leukocytes from the resultant cattle were able to inhibit the *P. hameolytica* leukotoxin-induced cytotoxicity [102]. 

## 7. Ethics, Regulations, and Social Acceptance of Genome-Edited Livestock Products

Manipulation of genomes in farm animals is becoming a lucrative and materialistic alternative. However, problems such as regulatory legislation, market pay off, and performance acceptability of users are still unresolved. The societal attitude towards genome-edited animal products worldwide will depend on whether the handling was carried out with due regard to the ethical value of target customer community and the issue of animal welfare [103]. The resulting foods after genome-edited livestock should appear on the commercial market ready to be distributed to consumers immediately, with the required legislative approval of a country. However, the contested factors linked to genetically modified (GM) as well as cloned animals suggest that food derived from genome-edited animals is socially rejected or unable to accept it. Psychological studies have shown that several factors such as the consumer’s perceived risks of the consumer, the recognized benefits and/or the confidence in regulatory legislation, can cause the acceptability of GM animals by researchers [104]. The public’s skeptical view of GMOs (GM organisms) is partly related to the degree of skepticism of both researchers and state policymakers [105]. Off-target mutagenesis seems to be a big problem due to the widespread use of the CRISPR/Cas9 tool. In relation to the breeding of disease resistant animals, the problem is of more serious concern. The debate on closed animals, stimulated by so many tests, prompted the study of off-target mutation. Exploration of off-target mutations tends to be crucial from the point of view of animal health and ethics to the broader implications of genome-editing techniques in animal breeding [103]. Therefore, in addition to scientific ethics, ethical concerns of animal health and welfare to minimize the imminence of off-target mutations are required to enhance public understanding. This could advocate the social acceptance of genome-edited livestock products gradually.

## 8. Potentials and Prospects of CRISPR/Cas9 Technology in Livestock Production

The CRISPR/Cas9 exhibits the potentials for substantial improvement over the gene-editing technologies in the ease of application, speed, efficiency, and cost involved. The genome-editing technique has been extensively used for elucidating the gene function in disease pathogenesis and host immune responses. Indeed, the CRISPR/Cas9-based correction of gene mutation in a mouse model of human disease and the primary adult stem cells derived from patients suffering monogenic hereditary defects are being considered the cornerstones for future gene therapy technique [9]. The CRISPR/Cas9-mediated knockout libraries could also potentially be applied to target regions of interest in the noncoding regulatory segment of the genome, such as promoter and enhancer. Moreover, the application of CRISPR/Cas9 in genome-wide studies will facilitate the holistic screening of disease resistance markers. The CRISPR/Cas9 technique would also enable a much wider range of modifications, for instance, gene knockout, base-pair substitution, targeted insertion/deletion of larger genomic regions, and modulation of gene expression [8]. Despite many challenges, remarkable advancements in the field of gene therapy and CRISPR/Cas9-based genome editing technique have been observed in recent years, which paves the way for the development of sustainable disease control strategies for humans, crops, fish, and livestock. Though unlikely in crops, where the whole population can rapidly be replaced, the application of genome editing in the livestock population is a more complex and time-consuming process. Moreover, increasing the efficiency of the CRISPR/Cas9-based repairing process, particularly to increase the rate of gene correction and reduce resultant off-target effects and the development of more effective delivery methods would require for its wider application. 

## 9. Conclusions

Raising immunocompetent healthy livestock is crucial for the sustainable production of safe food. Understanding the genetics behind the host immunocompetence to infectious pathogens is the key to improve the level of disease resistance through molecular breeding approaches. Exploring the variation of host innate immunocompetence to economically important infectious diseases among indigenous, exotic, and cross-bred animals of the same species can be of important starting point toward estimating the genetic components of disease resistance phenotype. The cutting-edge techniques for immunological and molecular genetics study may create a direct linkage between disease-resistant phenotype and the host genotype. Continuous advancement of open-source in silico omics tools will identify the potential genomic marker, the target for genome editing for disease resistance in livestock. The application of modern reproductive biotechnology, such as CRISPR/Cas9 mediated genome editing, is a breakthrough tool for improving disease resistance in livestock due to its high precision. Minimizing the risk of off-target mutations would restore the animal welfare standard and increase consumer acceptance of food products derived from genome-edited livestock.

## Figures and Tables

**Table 1:** Bioinformatic tools or database commonly used for transcriptomics or immunogenomics studies aiming to identify the key regulatory genes in animals.

Bioinformatics Tools/Databases	Potential Implications	References
‘Bowtie’, ‘msa’	Sequence-read alignment,	https://cran.r-project.org/
R/Bioconductor, limma, DESeq2	Differential gene expression analyses	https://cran.r-project.org/
GSEA-Gene Set Enrichment Analysis	Gene set enrichment analysis	[41] https://www.gsea-msigdb.org/gsea/index.jsp
DAVID	Gene ontology and pathway analysis	[42] https://david.ncifcrf.gov/
KEEG-Kyoto Encyclopedia of Genes and Genomes	Gene ontology and pathway analysis	https://www.genome.jp/kegg/
InnateDB	Database for gene ontology, pathway analysis and prediction interactome	[43] https://www.innatedb.com/
REACTOME	Database for gene ontology and pathway analysis	[44] https://reactome.org/
QTLdb	Database of quantitative trait loci of animals	[39] https://www.animalgenome.org/cgi-bin/QTLdb/index
BovineMine	Annotation and functions of gene	[45] http://128.206.116.13:8080/bovinemine/begin.do
bioDBnet-Biological database network	Interconnected access to many types of biological databases, conversion of gene or protein identifies	[46] https://biodbnet-abcc.ncifcrf.gov/
STRING	Functional protein association network analysis and visualization	[47] https://string-db.org/
NetworkAnalyst	Co-regulatory gene or protein network analysis and visualization	[48] https://www.networkanalyst.ca/
WGCNA	Weighted gene co-expression network analysis	[49] https://cran.r-project.org/
Cytoscape	Creation and visualization gene network	[50] https://cytoscape.org/

**Table 2:** Reported application of genome editing techniques for disease resistance livestock breeding.

Species	Disease	Targets of Genome Modification	Reference
Goat	Mastitis	Lysozyme (human)	[88]
Cattle	Mastitis	Lysostaphin (*Staphylococcus simulans*)	[84]
	Enzootic pneumonia	Cluster of differentiation 18 (CD18)	[89]
	Tuberculosis	The natural resistance to infection with intracellular pathogens 1 (NRAMP1) gene	[90]
Pigs	African swine fever	RELA	[86]
	PRRS	Histone deacetylase HDAC6	[91]
		Cluster of differentiation 163 (CD163)	[92,93]
